# Surgical treatment of multiple magnet ingestion by children: A single-center experience from China

**DOI:** 10.5339/qmj.2024.4

**Published:** 2024-03-26

**Authors:** Chen Zhang, Xiao-Wen Zhang, Shao-Mei Liu, Ke-Hua Huang, Zi-Min Chen

**Affiliations:** 1 Department of General Surgery, Shenzhen Children’s Hospital, China Medical University, Shenzhen 518038, China Email: ziminchen@126.com

**Keywords:** Children, multiple magnet ingestion, perforation, surgery

## Abstract

Background: The incidence of magnet ingestion in children has escalated concurrent with the rise in popularity of magnetic playthings, bearing the capacity to induce substantial morbidity.

Aim: The objective of this study was to encapsulate our accumulated expertise in handling pediatric cases featuring multiple magnetic foreign bodies within the gastrointestinal tract sometimes necessitating surgical intervention, as well as to formulate a clinical management algorithm.

Methods: This was a retrospective review of patients with multiple magnetic foreign bodies in the digestive tract, admitted to Shenzhen Children’s Hospital, between January 2018 and December 2022.

Results: A total of 100 cases were included in this study, including 66 males and 34 females. The main clinical manifestation ns were abdominal pain and vomiting. All patients had abdominal x-ray, all of which indicated foreign bodies in the digestive tract. 33 patients had to undergo a surgical intervention. Among these cases, the gastrointestinal complications occurred in 31 patients, including gastric rupture (n = 9), intestinal obstruction (n = 11) and intestinal perforation (n = 30). Postoperative intestinal obstruction occurred in 6 children. There was no statistical significant difference in age and gender between the Surgical group and Non-surgical group, but the Surgical group had a higher number of magnets ([7.5(2-44) vs 4(2-20)], p = 0.009), a longer interval between time of misingestion to clinical visit ([48(7.2-480) vs 5(2-336)]hours, p < 0.001), and a longer length of hospital stay ([10(6-19) vs 2(1-8)]days, p < 0.001).

Conclusions: Multiple magnet ingestion in children can lead to serious complications and carry severe risks. Timely diagnosis and effective treatment are crucial for managing such patients.

## Introduction

**Figure 1.**
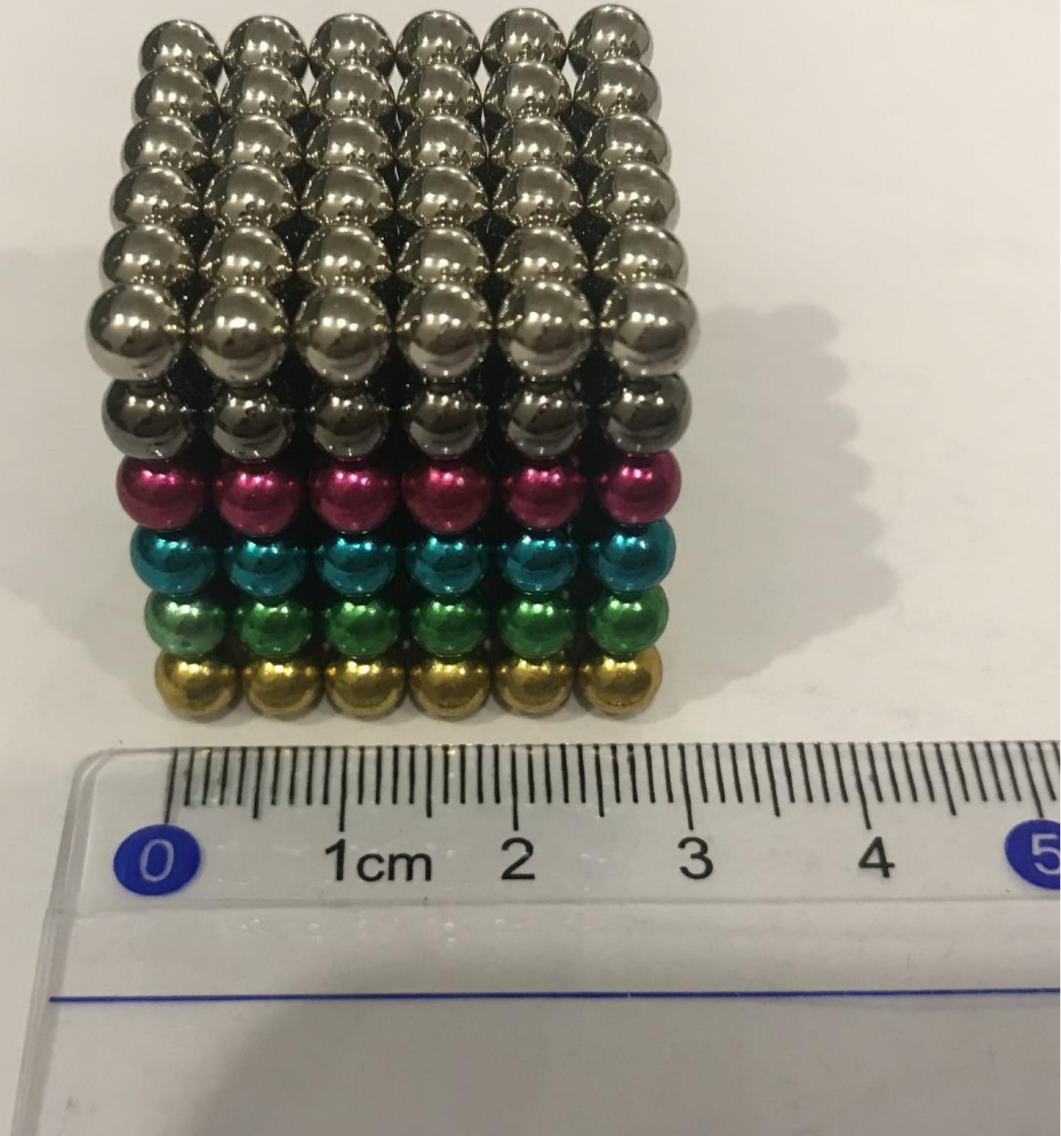
Photograph of Buckballs.

Foreign bodies in the digestive tract of children have emerged as a prevalent global issue, ranking high among unintentional injuries.^1^ The array of objects unintentionally swallowed encompasses a range of everyday items, including coins, small toys, batteries, and magnets. Managing such incidents requires a multifaceted approach involving conservative treatment, emergency endoscopy, and surgical intervention.^2^ It’s noteworthy that a mere 1% of cases necessitate surgical intervention due to foreign body retention or related complications.^3^ In recent times, there has been a noticeable surge in the incidence of magnet ingestion, partly attributable to the soaring popularity of a mesmerizing toy named “Buckball” (Figure 1). These tiny spheres, measuring approximately 5 mm in diameter, possess formidable magnetic properties. Once accidentally ingested, they could steadily attract one another, even though six layers of the bowel wall apart.^4^ After the restrictions on “Buckball” were lifted in the United States in 2016, the number of people undergoing surgery due to accidental ingestion of magnetic beads has increased significantly.^5^ Unfortunately, the current body of literature on the surgical treatment of multiple magnetic foreign bodies remains limited, lacking in robust, high-quality studies. The majority of published studies in this realm predominantly adopt a descriptive approach. In light of this, we present a large case series of magnet ingestion in pediatric patients, aiming to provide clinical experience in early diagnosis, rational management options, and preventive measures.

## Materials and Methods

We meticulously examined the medical records of all patients who had ingested multiple magnets at Shenzhen Children’s Hospital, spanning from January 2018 to December 2022. The diagnoses for these patients were predicated upon their medical history, results of imaging examinations, and operative records. The inclusion criteria required participants to be aged 14 years or younger and to have ingested two or more magnets. Exclusion criteria encompassed individuals older than 14 years, solitary magnet ingestion, and cases with incomplete medical documentation. Subsequently, comprehensive clinical data were collected from the included patients, encompassing demographic information, primary complaints, symptoms, physical manifestations, imaging findings, the duration between ingestion and admission, surgical procedures, outcomes, and follow-up details. The patients were divided into two groups based on whether they underwent surgery, namely the Surgical group and the Non-surgical group. Descriptive count data with normal distribution are represented by means ± standard deviation and the differences between two groups were evaluated by independent sample t test. While, data with non-normal distribution are represented by median (minimum-maximum), and the differences were evaluated by Mann-Whitney U test. The difference of gender distribution between two groups was evaluated by chi-square test. All reported p values are two-tailed, and statistical significance was set at P < 0.05. Statistical analysis was conducted employing IBM SPSS Statistics 23.0 (IBM Corp., Armonk, NY). This study obtained approval from the Medical Ethics Committee of the Shenzhen Children’s Hospital (approval number 202200402).

## Result

### General Data

**Figure 2.**
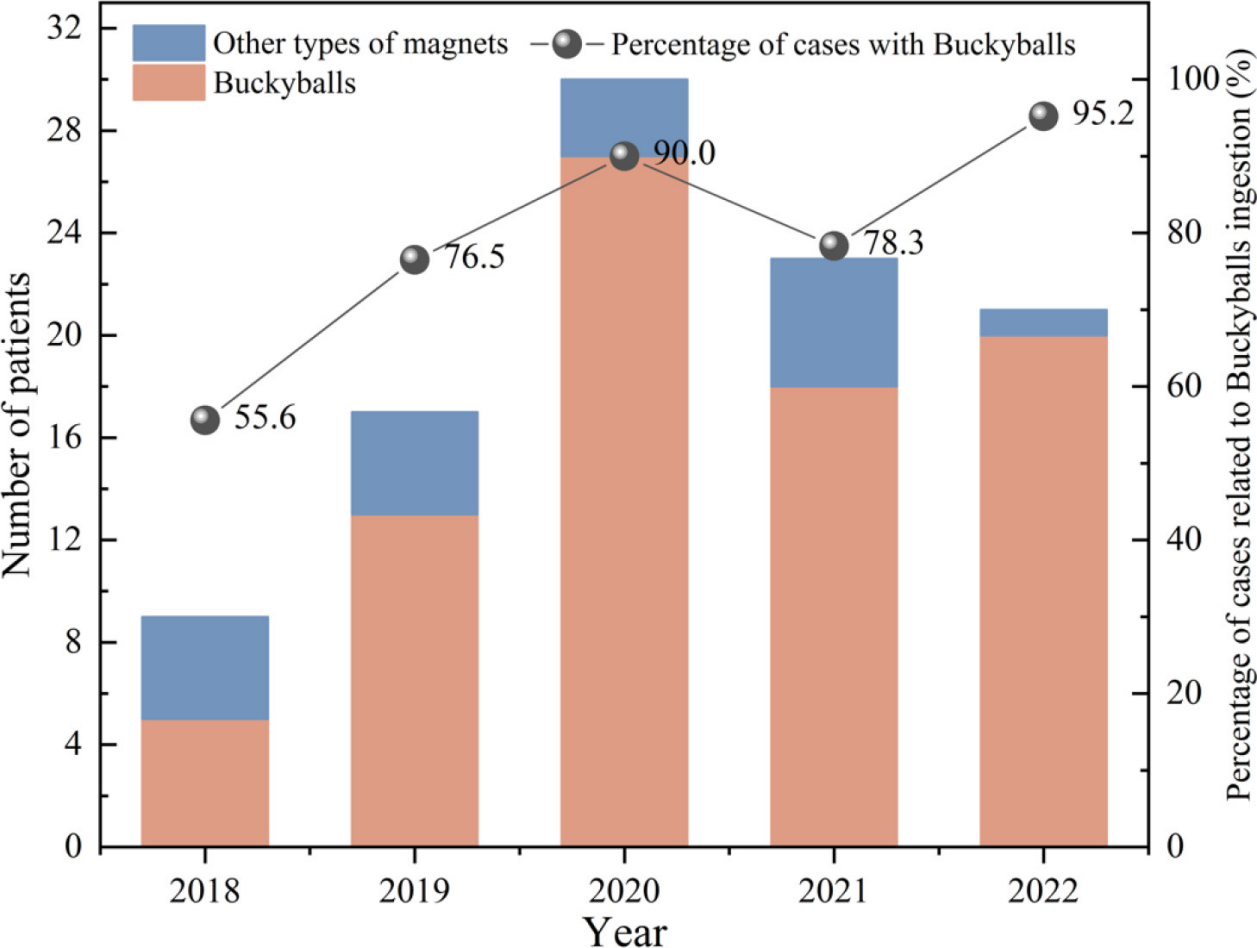
The number of patients with multiple magnets ingestion and the proportion of patients with Buckyball ingestion at Shenzhen Children’s Hospital from 2018 to 2022.

Between January 2018 and December 2022, Shenzhen Children’s Hospital received a total of 100 patients with multiple magnetic foreign bodies, out of which 33 had to undergo a surgical intervention. The magnetic foreign bodies encompassed 83 cases of Buckyballs and 17 cases involving other types of magnets (Table 1). The percentage of patients who had ingested Buckyballs has shown a general increasing trend over the years (Figure 2). The gender distribution exhibited a ratio of 4.9 males to every female. The age range spanned from 0.6 years old to 12 years old, with a median age of 4.8 years old. Compared with the Non-surgical group, there was no significant difference in age and gender (Table 2).

### Clinical Manifestations

The number of ingested magnetic foreign bodies ranged from 2 to 44, with a median of 4.0. In the Non-surgical group, all children or parents were able to provide a comprehensive account of foreign body ingestion during the medical consultation. In the Surgical group, only 21 patients (63.6%) were able to provide a detailed history of foreign body ingestion. Compared to the Non-surgical group, the Surgical group had a longer interval between time of misingestion to clinical visit and higher number of magnets (Table 2).

Notably, within the Surgical group, 29 cases (84.8%) exhibited symptomatic manifestations, which included 20 cases of abdominal pain, 22 cases of vomiting, and 3 cases of fever. Conversely, in the Non-surgical group, only 12 cases (18.0%) exhibited clinical symptoms, including 9 with abdominal pain and 3 with vomiting. In terms of physical examination, there were 23 cases (69.7%) with positive signs in the Surgical group, including abdominal bloating in 10 cases, only abdominal tenderness in 15 cases, and peritoneal irritation signs in 7 cases. In the Non-surgical group, only 3 patients (4.5%) presented with only abdominal tenderness (Table 1).

### Image Analysis

**Figure 3.**
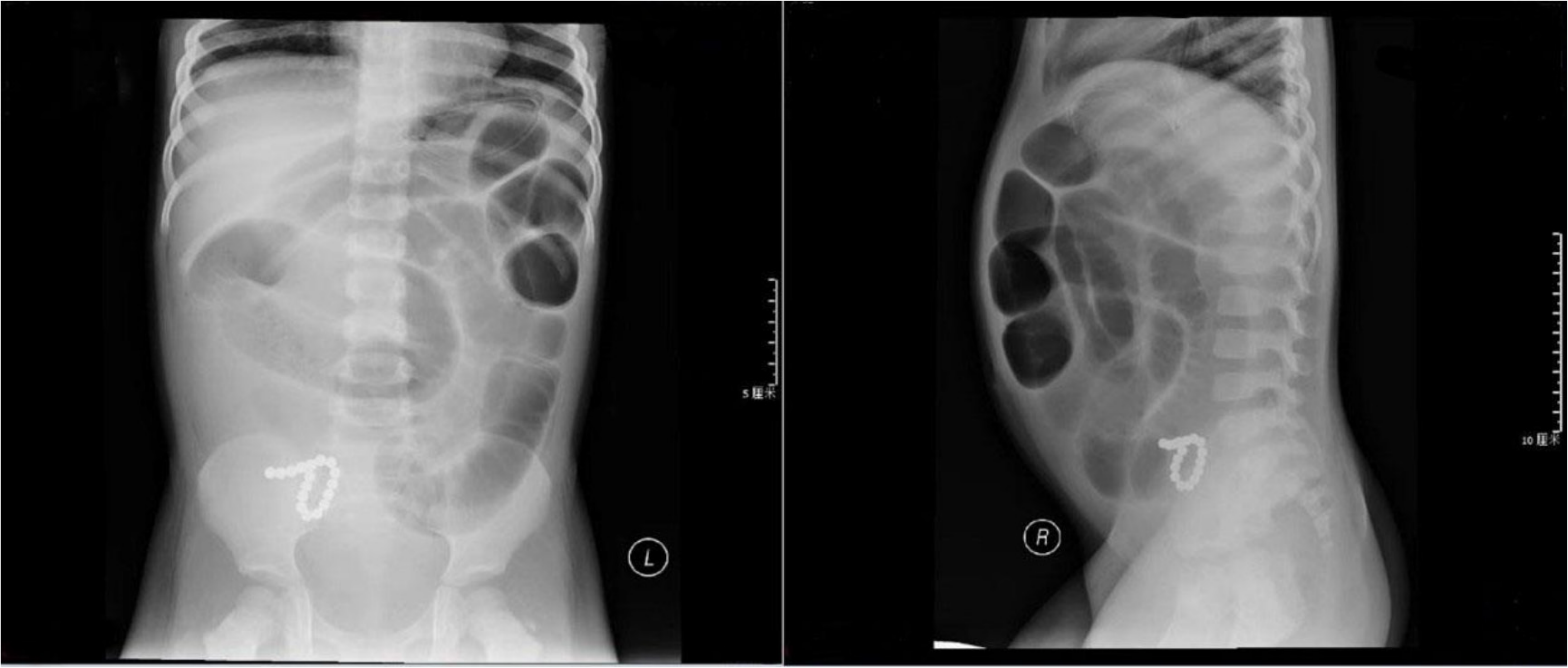
Anterior and lateral X-ray views of a patient who ingested a string of magnetic beads.

**Figure 4.**
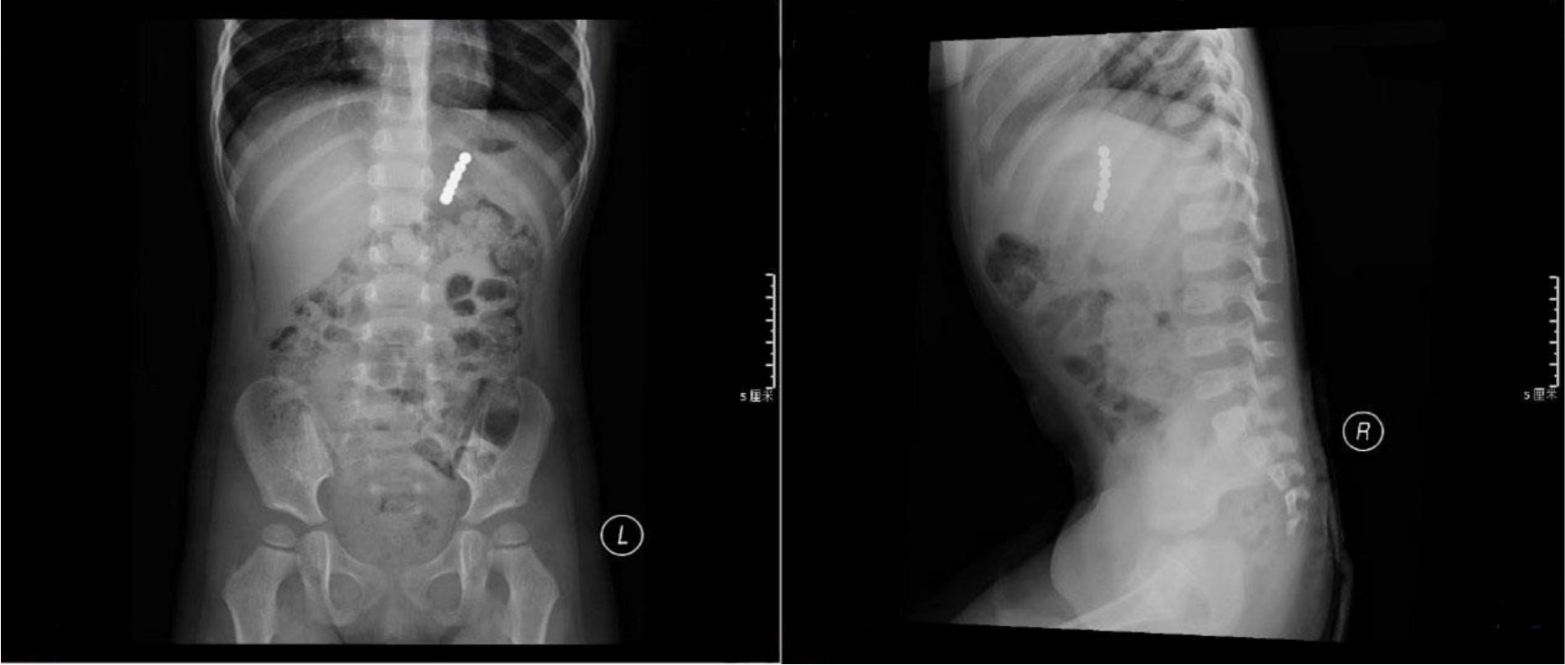
Anterior and lateral X-ray views of a patient who ingested a small number of magnetic beads.

All cases underwent plain abdominal radiography, which confirmed the presence of multiple magnetic foreign bodies. The radiographic depiction of two examples of ingested magnets is showcased in Figures 3 and 4. Due to suspected gastrointestinal complications, 9 cases underwent ultrasonography, revealing intestinal obstruction in 7 cases and gastrointestinal perforation in 2 cases. Furthermore, 5 cases underwent CT examinations, revealing intestinal obstruction in 5 cases, gastrointestinal perforation in 4 cases. Subsequently, all 13 patients underwent surgery, with gastrointestinal obstruction and perforation confirmed in each case.

### Clinical Management and Prognosis

According to the radiographs, in 30 cases, the magnets were located in the upper digestive tract, so gastroduodenoscopies were performed. Gastrointestinal perforation was found in 6 cases, and a follow-up radiograph revealed the presence of foreign objects in the abdomen. Subsequently, the patients were transferred to surgery. The remaining 24 cases had all foreign bodies successfully removed, and they suffered no gastrointestinal complications. 70 cases’ magnets were located in the lower digestive tract. Among them, 27 patients exhibited signs of severe gastrointestinal complications and had to undergo surgical intervention. For patients without signs of severe gastrointestinal complications, conservative treatment was employed, such as series X-rays taken 6-12 hours apart and close observation. All patients receiving conservative treatment successfully passed the magnets without experiencing serious gastrointestinal complications.

**Figure 5.**
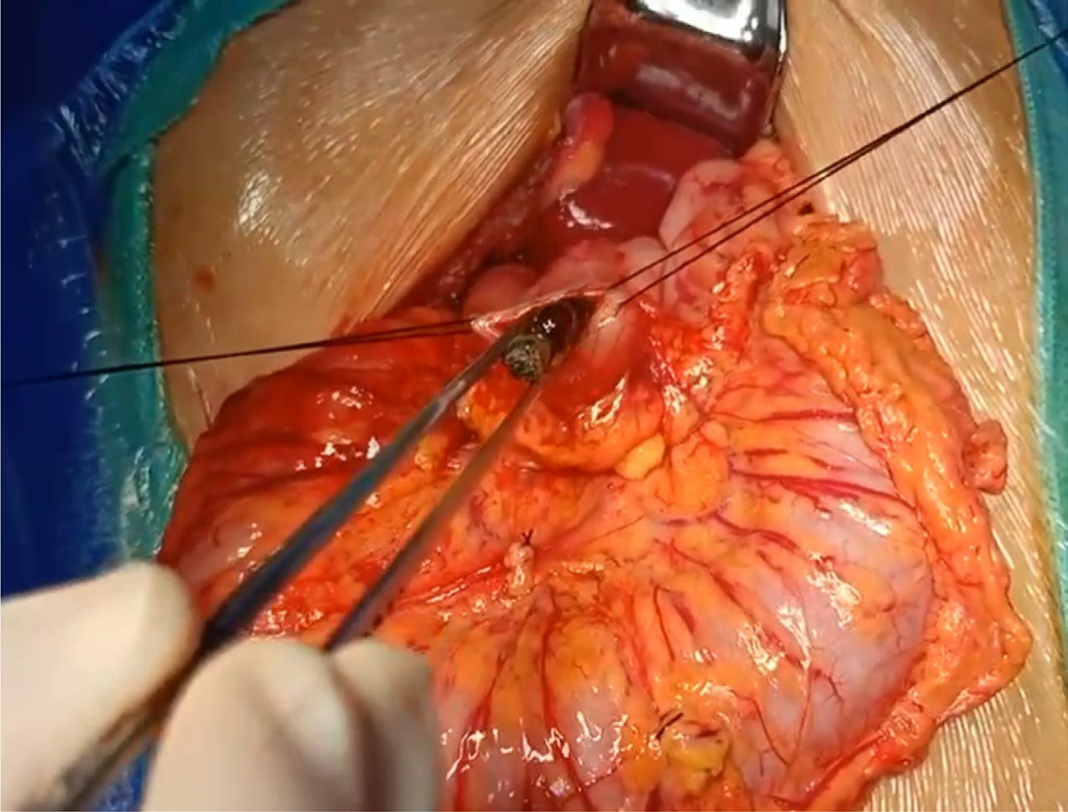
Procedure for magnetic beads removal.

**Figure 6.**
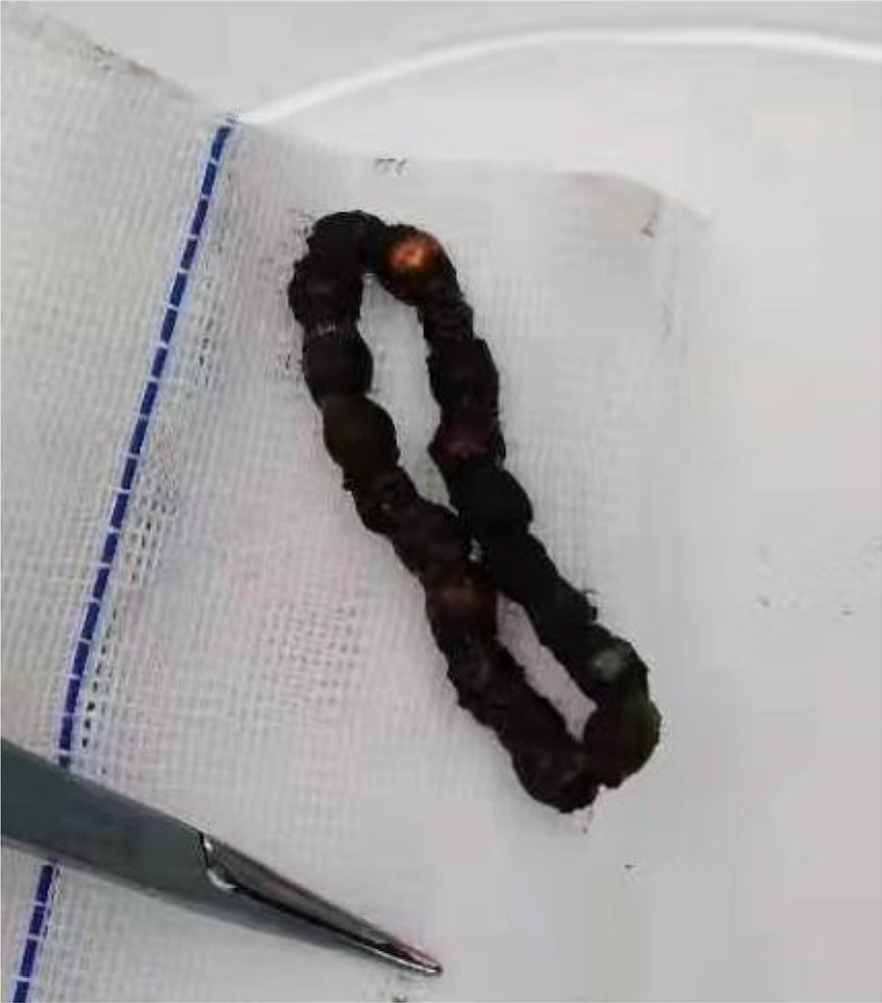
19 magnetic beads with tissue in between.

All cases in the Surgical group ultimately underwent laparotomy (Table 3). Out of these, 31 individuals experienced perforation within their digestive tracts, with the number of gastrointestinal perforations ranging from 0 to 10 and a median of 2.9. Notably, the small intestine emerged as the most frequent site for magnet adhesion. Other gastrointestinal complications included intestinal obstruction (11 cases), intestinal adhesion (25 cases), intestinal volvulus (1 case), and intestinal necrosis (1 case). The primary surgical approach entailed the removal of the foreign body through the original perforation site, followed by perforation repair (Figures 5 and 6). In cases where the foreign body could not be successfully extracted, an incision was made in the digestive tract to facilitate its removal, followed by subsequent repair. In 1 instance, due to the proximity of the foreign body to the appendix, the appendix was incised to extract the foreign object, after which the appendix itself was removed. 6 cases initially underwent endoscopic treatment, which was subsequently switched to surgical intervention due to perforation or the inability to completely extract all foreign bodies. Additionally, 6 cases initially began with laparoscopy but were later transitioned to open laparotomy. The specific reasons for this change included challenges in locating the foreign body, suspicion of intestinal necrosis, significant abdominal adhesions, and evident intestinal dilation.

All patients recovered uneventfully and were discharged home. Compared to the Non-surgical group, the Surgical group had a significantly longer length of hospital stay (Table 2). Remarkably, during a 6-month follow-up period, 6 children encountered episodes of intestinal obstruction, yet all instances were effectively resolved through conservative therapeutic measures. Encouragingly, there were no reported cases of repeated foreign body ingestion.

## Discussion

Children swallowing magnets is becoming a serious problem in public health that we need to pay attention to. Previous studies have shown that more and more children around the world are accidentally eating objects with magnets, and this problem keeps getting worse every year in many countries.^6,7^ This worrying trend is mainly because of the popularity of magnetic toys such as Buckyball. They are made of neodymium magnets, and are at least 30 to 50 times more powerful than traditional magnets. After multiple magnets were swallowed, they attracted each other tightly, resulting in local compression of the intestinal wall, ischemia, necrosis, perforation, peritonitis, internal fistula, intestinal obstruction and even death.^8^ In the study of Zheng Y et al, 45.1% of children who accidentally ate multiple magnets needed surgery.^9^ In addition, surgery exposing children to not only short and medium-term risks, such as anaesthetic complications and anastomotic leaks, but also the lifelong risks of subsequent complications, particularly adhesional bowel obstruction. Around 2010, Chinese sellers introduced Buckballs as educational toys, and kids loved them because they could learn while having fun. However, the attendant perils and deleterious ramifications of Buckballs have been distressingly overlooked.^10^ As the data gleaned from our meticulous analysis aptly illustrate, the patient tally continued its unrelenting ascent until 2020. Interestingly, after that, there was a noticeable decrease in the number of cases each year, which is a good sign. This decrease may be attributed to the increased awareness about the dangers of Buckballs. Nevertheless, we still cannot underestimate the dangers of Buckballs.

Within the 100 cases comprising this cohort, the prevalence of males outweighs that of females considerably, constituting a substantial 66.0%. This figure is close to the male-to-female ratio observed in prior investigations, which typically stood at approximately 2:1.^11^ This discrepancy can be attributed, in part, to the vivacious and mischievous disposition often associated with boys.^1^ Moreover, it is plausible that divergent approaches to parental guidance between the genders also play a role.^1^ Additionally, the constrained sample size within our study could be a contributing factor. In terms of age distribution, School-age children accounted for the highest proportion in this study. This may be attributed to factors such as the strong curiosity of school-age children and a lack of relevant safety knowledge.

In our study, the symptoms and signs in patients were mostly atypical, such as abdominal pain, vomiting, and abdominal tenderness. Similar circumstances have been reported by Zefov V et al.^12^ Furthermore, some children or parents may not be able to provide a comprehensive account of foreign body ingestion during the medical consultation, which makes early diagnosis difficult.^7,13^ Therefore, clinicians need to handle patients suspected of ingesting magnets with extra caution. Perforation and obstruction were the most common gastrointestinal complications in this study, necessitating surgical intervention. Through comparative analysis, we found that the number of ingested magnets and the interval between time of misingestion to clinical visit may be associated with the occurrence of these complications.

In the current investigation, the diagnosis of all patients was accomplished through abdominal radiography. Employing orthogonal radiography aids in ascertaining both the quantity and placement of magnets, along with detecting potential complications such as intestinal obstruction.^6,9^ Antecedent studies have revealed the limitations of X-ray imaging in distinguishing magnets from other foreign objects, such as metallic items (e.g., coins) and inorganic substances (e.g., pearls).^14^ In cases of perforation, accurate diagnosis can prove elusive. However, for non-radiopaque foreign bodies like magnets, we still recommend abdominal X-ray as the preferred examination. The numerous merits of abdominal sonography, including its non-invasive attributes, exceptional portability, and the assessment of gastrointestinal complications.^1^ Nonetheless, its diagnostic precision is dependable upon the expertise and experience of the physician.^15^ CT imaging has the capability to identify intestinal obstruction, perforation, abdominal inflammation, and other associated complications. However, the contentious aspect surrounding CT employment in the diagnosis of digestive tract foreign bodies stems from its considerable cost. Therefore, doctors need to make choices based on the actual circumstances when further examining gastrointestinal complications.

**Figure 7.**
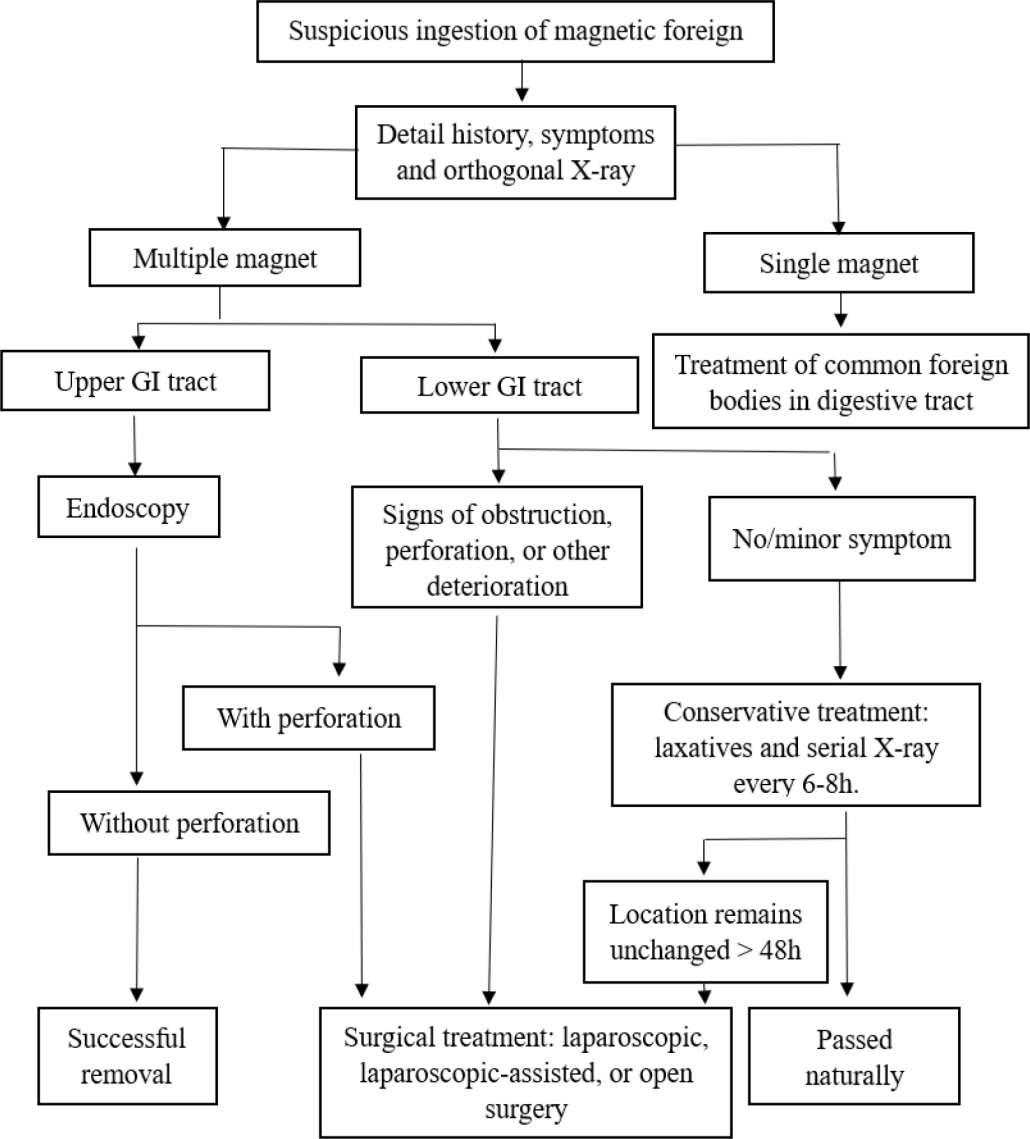
Suggested algorithm for management of multiple magnet ingestions in the pediatric population.

The proper management strategies for cases involving the ingestion of multiple magnets bear profound significance in enhancing patients’ overall outcomes. In previous studies, an estimated fifty percent of the children who inadvertently ingested multiple magnets necessitated surgical intervention.^9^ Such surgical procedures predominantly encompass laparoscopic surgery and laparotomy.^15,16^ The former is the traditional surgical approach, suitable for all gastrointestinal foreign bodies. The latter, as indicated by Cai DT et al., can be employed for managing patients who have ingested foreign bodies for more than 2 weeks and exhibit milder clinical symptoms and signs, with the foreign body located in the small intestine.^10^ In instances of laparoscopic surgery, the metallic tips of laparoscopic instruments may facilitate the identification of magnets.^17^ However, the mutual attraction between surgical instruments and magnets can exacerbate the operative complexity, extend the duration of the procedure, and potentially result in intestinal harm, minute perforations, and mesenteric lesions.^10^ Within our department, initially, 6 cases underwent laparoscopic surgery, which was later switched to laparotomy due to various reasons. Consequently, we still advocate for laparotomy as the primary choice. When confronted with the scenario of multiple magnet ingestions transpiring within the stomach or esophagus, endoscopic extraction may mitigate the necessity for surgical intervention in select cases, provided that the timeframe since ingestion does not surpass 12 hours.^9^ Prudent manipulation becomes paramount in this context, as imprudent handling runs the risk of gut perforation and subsequent leakage.^18^ In situations where children exhibit no severe symptoms and magnet migration beyond the stomach is suspected without signs of obstruction or perforation, a conservative approach can be adopted.^9,19^ This entails the administration of laxatives and the acquisition of sequential plain radiographs to monitor magnet movement while awaiting natural passage. Consequently, drawing upon the existing body of literature alongside our own clinical experiences,^15,20-26^ a flow chart was introduced for the management of multiple magnetic ingestions in the pediatric population (Figure 7).

In the United States, the overturning of legislation pertaining to the ban of magnet products in 2016 resulted in a notable resurgence of magnet ingestion cases.^5^ This occurrence underscores the considerable impact that shifts in national policy can exert on the incidence of multiple magnet ingestions. In China, magnetic toys, such as Buckballs, are readily obtainable by children through physical stores and online platforms.^21^ Despite the presence of warning signs, evidence suggests their limited efficacy in deterring magnet ingestion.^7,22^ Consequently, the implementation of more stringent policies becomes imperative in safeguarding children from acquiring products containing high-power magnets. Simultaneously, the role of parental supervision emerges as a pivotal factor in mitigating injuries.

The study is limited by the retrospective nature of the data collection, relying on complete, and accurate records in patient case notes. The factor create the potential for selection bias. Secondly, while the data were collected from a tertiary center, it is important to acknowledge that the study sample size comprises only 100 cases.

## Conclusion

The initial presentations of multiple magnet ingestion in pediatric cases often lack typical characteristics, but can result in severe complications, especially if their removal is delayed or if a large number of magnets are ingested. In our study sample, approximately one-third of patients who ingested multiple magnets experienced severe gastrointestinal complications, necessitating surgical intervention. It is imperative for pediatricians to maintain constant vigilance regarding the potential hazards associated with such conditions. Moreover, there is an urgent demand for more stringent regulatory measures to deter children from acquiring perilous magnetic products.

## Funding

This work was supported by Guangdong High-level Hospital Construction Fund and Shenzhen Health and Medical Elite Talent Development Program (2022XKG066).

## Figures and Tables

**Table 1.** Clinical features of patients with multiple magnetic foreign body ingestion.

	**Surgery (N = 33)**	**No surgery (N = 67)**	**Total**
Types of foreign bodies			
Buckyball	27 (81.8%)	56 (83.6%)	83 (83.0%)
Others	6 (18.2.0%)	11 (16.4%)	17 (17.0%)
Age			
Infancy period (0-1 year old)	2 (6.1%)	1 (1.5%)	3 (3.0%)
Toddler period (1-3 years old )	12 (36.4%)	7 (10.5%)	19 (19.0%)
Preschool period (3-5 years old)	8 (24.2%)	22 (32.8%)	30 (30.0%)
School-age period (5-13years old)	11 (33.3%)	37 (55.2%)	48 (48.0%)
Symptoms			
No	4 (12.1%)	55 (82.1%)	59 (59.0%)
Yes	29 (87.9%)	12 (17.9%)	41 (41.0%)
Abdominal pain	20 (60.1%)	9 (13.4%)	29 (29.0%)
Vomiting	22 (66.7%)	4 (6.0%)	26 (26.0%)
Fever	3 (9.1%)	0	3 (3.0%)
Abdominal distension	3 (9.1%)	0	3 (3.0%)
Decreased defecation	4 (12.1%)	0	4 (4.0%)
Signs			
No	10 (30.3%)	64 (95.5%)	74 (74.0%)
Yes	23 (69.7%)	3 (4.5%)	26 (26.0%)
Abdominal bloating	10 (30.3%)	0	10 (10.0%)
Abdominal tenderness only	15 (45.5%)	3 (4.5%)	18 (18.0%)
Peritoneal irritation signs	7 (21.2%)	0	7 (7.0%)

**Table 2.** Difference between the Surgical and the Non-surgical group.

	**Surgery (N = 33)**	**No surgery (N = 67)**	**P**
Age (years)	4.1 ± 2.3	5.4 ± 2.3	0.07
Gender			0.148
Male	25	41	
Female	8	26	
The number of magnets	7.5 (2-44)	4 (2-20)	0.009
Interval between time of misingestion to clinical visit (hours)	48 (7.2-480)	5 (2-336)	< 0.001
Length of hospital stay(days)	10 (6-19)	2 (1-8)	< 0.001

**Table 3.** Summary of surgical intervention for all patients.

	**Number**	**Proportion (%)**
Surgical intervention		
Laparotomy	21	63.6
Endoscopy converted to laparotomy	6	18.2
Laparoscopy converted to laparotomy	6	18.2
Location of ingested foreign body		
Small intestine	12	36.4
Small intestine + cecum	4	12.1
Stomach + duodenum	4	12.1
Stomach + small intestine	4	12.1
Stomach + colon	3	9.1
Small intestine + colon	3	9.1
Colon	1	3.0
Cecum	1	3.0
Rectum	1	3.0
Location of perforation		
None	2	6.1
Small intestine - small intestine	19	57.6
Stomach - small intestine - transverse colon	1	3.0
Small intestine - sigmoid colon	3	9.1
Small intestine - lleocecal region	2	6.1
Stomach - small intestine	4	12.1
Small intestine - ileocecal region - transverse colon	1	3.0
Stomach - Duodenum	1	3.0
Stomach	1	3.0
Stomach - transverse colon	1	3.0
